# Are There Racial Disparities in Knee Symptoms and Articular Cartilage Damage in Patients Presenting for Arthroscopic Partial Meniscectomy?

**DOI:** 10.2106/JBJS.OA.21.00130

**Published:** 2022-09-22

**Authors:** Christa L. Wentt, Lutul D. Farrow, Joshua S. Everhart, Kurt P. Spindler, Morgan H. Jones

**Affiliations:** 1 Howard University Hospital, Washington, DC; 2 Cleveland Clinic Orthopaedic and Rheumatology Institute, Cleveland, Ohio; 3 Indiana University Health, Indianapolis, Indiana; 4 Department of Orthopaedic Surgery, Brigham and Women’s Hospital, Boston, Massachusetts

## Abstract

The purpose of the present study was to examine whether Black patients presenting for arthroscopic partial meniscectomy (APM) have worse baseline knee pain, worse knee function, and greater articular cartilage damage than White patients.

**Methods:**

A cohort of 3,086 patients (84% of whom were White; 13%, Black; and 3%, other race, with a median age of 53 years) who underwent APM were enrolled. Patients who underwent concomitant procedures and patients of undisclosed race or self-pay status were excluded. The associations of race with the preoperative Knee injury and Osteoarthritis Outcome Score (KOOS) for pain (KOOS-pain) and the KOOS-Physical Function Short Form (KOOS-function) and the intraoperative assessment of cartilage damage (highest modified Outerbridge grading) were determined by multivariate modeling with adjustment for age, sex, insurance status, years of education, smoking status, body mass index (BMI), meniscal tear location, and Veterans RAND 12-Item Health Survey Mental Component Summary (VR-12 MCS) score.

**Results:**

The 3 factors most strongly associated with worse KOOS-pain and KOOS-function were a lower VR-12 MCS score, increased BMI, and increased age. The 3 factors most strongly associated with higher-grade articular cartilage damage were increased age, increased BMI, and meniscal tear location. All of these factors had an unequal distribution between Black and White patients. After adjusting for confounding variables, the KOOS-pain score for Black patients was a mean of 2.6 points lower than that for White patients regardless of insurance status; the KOOS-function score for Black patients with commercial insurance was a mean of 2.4 points lower than that for White patients with commercial insurance but was not lower than that for Black patients on Medicare. Compared with commercially insured White patients, commercially insured Black patients had 1.4-fold greater odds of having higher-grade articular damage, and no difference in risk was detected among Medicare-insured Black patients.

**Conclusions:**

There are clinically important differences in the distribution of risk factors between Black and White patients presenting for APM regarding several factors associated with worse knee pain, knee function, and greater articular cartilage damage. When controlling for these confounding factors, a significant, but not clinically relevant, racial disparity remained with respect to knee pain, knee function, and cartilage damage. Two of the 3 major risk factors for all 3 included age and BMI. The third factor for knee pain and function was mental health, and the location of a meniscal tear was the third factor for articular cartilage damage.

**Level of Evidence:**

Prognostic Level III. See Instructions for Authors for a complete description of levels of evidence.

Racial disparities exist in multiple areas of surgery^1-4^. Several studies have evaluated racial disparities in orthopaedic surgery, particularly regarding joint arthroplasty and spine surgery, in the United States. Access to care and patient outcomes differ between White and Black patients^5-19^. White-Black disparities in total elbow arthroplasty utilization have increased over time^16^. The use of total knee and hip arthroplasty has been disparately lower in Black patients than White patients, despite a higher prevalence of knee osteoarthritis and more complicated and severe disease at presentation among Black patients^15^. Disparities have also been reported between patients with different insurance payer types with respect to total joint arthroplasty and spine surgery^10,20^. Those studies did not evaluate patient-reported outcome measurements (PROMs) at baseline.

Because there are several national databases for spinal and arthroplasty procedures, much research has been done on disparities pertaining to these procedures, although these databases do not report PROMs. There is presently no equivalent database for procedures in sports medicine. Although some studies have evaluated race in their models^7^, there currently are no studies specifically evaluating for evidence of racial disparities in sports medicine surgery.

The objective of this study was to evaluate whether racial disparities exist with respect to knee pain, knee function, and concomitant articular cartilage damage among patients who undergo arthroscopic partial meniscectomy (APM). We hypothesized that Black patients seen for APM have worse knee pain, worse knee function, and greater articular cartilage damage than White patients after controlling for other relevant factors in multivariate analysis.

## Materials and Methods

### Participants

**Fig. 1:**
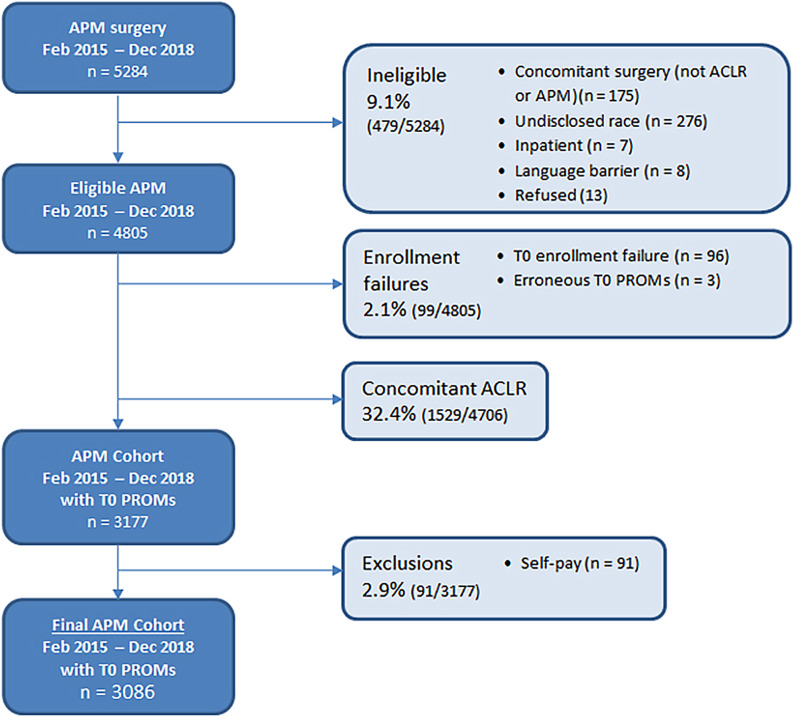
STROBE (STrengthening the Reporting of OBservational studies in Epidemiology) diagram. APM = arthroscopic partial meniscectomy, ACLR = anterior cruciate ligament reconstruction, T0 = time zero (baseline), and PROMs = patient-reported outcome measures.

The present study received institutional review board approval. All patients undergoing elective sports medicine and arthroscopic procedures at Cleveland Clinic are prospectively enrolled in the Outcomes Measurements Evaluations (OME) cohort^21^. OME is a secure data collection system developed at Cleveland Clinic to prospectively capture patient demographics, joint-specific and whole-body PROMs, quality-of-life (QoL) scores, physical examination findings, and intraoperative findings. The study population was selected from an initial, prospective cohort of 5,284 patients from February 2015 to December 2018 who underwent APM (Fig. 1). The primary exclusion was concomitant anterior cruciate ligament reconstruction (ACLR) in 1,529 (32.5%) of 4,706 patients. Our enrollment failure rate was 2.1%. We excluded self-pay status (2.9%) to reach our final prospective data set of 3,086. Further details are provided in our STROBE (STrengthening the Reporting of OBservational studies in Epidemiology) figure (Fig. 1).

### Data Collection

OME data were exported to a secure Research Electronic Data Capture (REDCap) database^22,23^. Age, sex, body mass index (BMI), smoking status, PROMs, and intraoperative findings were collected as part of OME. Race and insurance status are standard elements in the electronic medical record (EMR), and therefore OME derived these data from the EMR. Race entries available in the EMR included White, Black, Asian, multiracial, Native American, other, and not listed; these entries are based on patient self-identification on intake forms. All races not reported as Black or White were classified as “other” for the purpose of this study.

All PROMs were completed prior to surgery. Preoperative knee pain was assessed with the Knee injury and Osteoarthritis Outcome Score (KOOS) for pain (KOOS-pain)^24^. Preoperative patient-reported knee function was assessed with the KOOS Physical Function Short Form (KOOS-function)^25^. Mental health was assessed using the Veterans RAND 12-Item Health Survey Mental Component Summary (VR-12 MCS) score^26^.

Intraoperative findings were entered by the surgeon into OME. Articular cartilage status was assessed according to the modified Outerbridge criteria^27^, which is used extensively in multicenter clinical studies, including the Multicenter Orthopaedic Outcomes Network (MOON). Normal cartilage was assigned grade 0; cartilage with soft indentation and/or superficial fissures or cracks, grade 1; cartilage with lesions involving <50% of cartilage depth, grade 2; cartilage with lesions involving a depth of ≥50% but not extending through subchondral bone, grade 3; and cartilage with lesions penetrating through the subchondral plate, grade 4^27^. The degree of articular cartilage damage was defined by the highest Outerbridge grade encountered within any knee compartment.

All patients underwent partial meniscectomy of the medial and/or the lateral meniscus. If a complete tear was encountered (tear extension from the center to the periphery of the meniscus, or extension through both the superior and inferior meniscal surfaces), the surgeon noted the location of the tear as medial, lateral, or both menisci. Intrasubstance degeneration was classified as no complete tear. Tear involvement of the meniscal root was also recorded.

### Statistical Analysis

A minimal clinically important difference (MCID) of 8 points^24^ was used for KOOS-pain and KOOS-function, and a true odds ratio (OR) of 1.5 was used for articular cartilage damage in an initial power analysis. The study was adequately powered (>80% power at alpha = 0.05) to compare outcomes between Black and White patients but was underpowered to compare the 85 patients of “other” race (3% of the study sample).

Normal variables were summarized with the mean and standard deviation (SD), and non-normal variables were summarized as the median and interquartile range (first and third quartiles; Q1 and Q3, respectively). Categorical variables were summarized as the frequency and percentage. Univariate relationships with race (White, Black, or other) were analyzed with analysis of variance (ANOVA; normal continuous variables), the Kruskal-Wallis test (non-normal continuous variables), and the Pearson chi-square test (categorical variables). Comparisons between groups were done with Tukey adjustments when the row variable was normally distributed and with the Benjamini-Hochberg method otherwise.

Multivariate linear regression was used to identify drivers of KOOS-pain and KOOS-function. A multivariate proportional-odds regression model was used to identify drivers of articular cartilage damage (Outerbridge grades I through IV). Linear regression and proportional-odds assumptions were verified graphically. All multivariate models included race, sex, insurance type, BMI, years of education, smoking status, baseline VR-12 MCS score, and meniscal tear characteristics. Interactions between race and insurance type, as well as between patient age and years of education, were tested. The race and insurance type interaction term was kept in all models, but the age and years of education interaction term was excluded if nonsignificant. To measure the relative importance of individual variables in the regression models, Akaike information criterion (AIC) importance plots were created. These plots illustrate the magnitude of change in the AIC value when the individual variable was removed from the statistical model. Finally, the overall quality of the regression models was assessed with validated, index adjusted R^2^, and the concordance index. P values of <0.05 were considered significant.

### Source of Funding

This study was not funded.

## Results

### Descriptive Statistics and Univariate Comparisons Between Races

There were 2,593 White patients (84%), 408 Black patients (13%), and 85 patients (3%) of “other” race, and they had a mean age of 53 years (Table I). A total of 40 surgeons at 14 sites entered their patient data. There were multiple clinically relevant and significant differences (p ≤ 0.001 for each comparison) in the distribution of sociodemographic variables between White and Black patients (Table I). White patients were older than Black patients (median, 54 and 50 years, respectively), had a lower mean BMI (30.3 and 32.4 kg/m^2^), were more likely to be male (58.5% and 49.0%), and were more likely to have a commercial insurance plan (80.5% and 65.9%). White patients were less likely to be current smokers (10.0%) than Black patients (16.7%). There was a difference in median years of education (14 years for White patients and 13 years for Black patients) and in the percentage of patients with <12 years of education (6.7% among White patients and 15.9% among Black patients).

**TABLE I:** Descriptive Statistics by Race*

Variable	Level	White (N = 2,593)	Black (N = 408)	Other (N = 85)	P Value		
					White Versus Black	White Versus Other	Black Versus Other
Age at surgery† *(yr)*		54.0 (44.0; 61.0)	50.0 (41.0; 57.0)	49.0 (36.0; 56.0)	<0.001	0.001	0.431
Sex *(no. [%])*					0.001	0.113	0.990
	Male	1,517 (58.5)	200 (49.0)	41 (48.2)			
	Female	1,076 (41.5)	208 (51.0)	44 (51.8)			
Insurance *(no. [%])*					<0.001	<0.001	0.580
	Commercial	2,088 (80.5)	269 (65.9)	61 (71.8)			
	Medicare	378 (14.6)	45 (11.0)	8 (9.4)			
	Medicaid	127 (4.90)	94 (23.0)	16 (18.8)			
Mean BMI (SD) *(kg/m*^*2*^*)*		30.3 (6.52)	32.4 (7.09)	29.8 (6.32)	<0.001	0.818	0.004
Length of education† *(yr)*		14.0 (12.0; 16.0)	13.0 (12.0; 15.0)	13.0 (12.0; 16.0)	<0.001	0.007	0.342
Length of education *(no. [%])*					<0.001	<0.001	0.621
	≥12 yr	2,419 (93.3)	343 (84.1)	69 (81.2)			
	<12 yr	174 (6.71)	65 (15.9)	16 (18.8)			
Smoking status *(no. [%])*					<0.001	0.725	0.684
	Never	1,602 (61.8)	255 (62.5)	54 (63.5)			
	Quit	731 (28.2)	85 (20.8)	21 (24.7)			
	Current	260 (10.0)	68 (16.7)	10 (11.8)			
Baseline VR-12 MCS†		55.9 (46.2; 62.0)	48.4 (38.4; 59.6)	50.5 (42.8; 59.3)	<0.001	0.024	0.203
Meniscal root tear *(no. [%])*					1.000	1.000	1.000
	No	2,478 (95.6)	394 (96.6)	82 (96.5)			
	Yes	115 (4.44)	14 (3.43)	3 (3.5)			
Complete meniscal tear location‡ *(no. [%])*					<0.001	<0.001	0.037
	None (incomplete tear)	739 (28.5)	140 (34.3)	40 (47.1)			
	Medial	1,290 (49.7)	151 (37.0)	18 (21.2)			
	Lateral	340 (13.1)	90 (22.1)	21 (24.7)			
	Both	224 (8.64)	27 (6.6)	6 (7.1)			
Baseline KOOS-pain†		47.2 (38.9; 61.1)	41.7 (25.0; 55.6)	47.2 (33.3; 61.1)	<0.001	0.323	0.022
Baseline KOOS-function†		58.0 (48.8; 64.7)	51.5 (38.0; 61.4)	56.0 (45.6; 64.7)	<0.001	0.345	0.015
Modified Outerbridge grade *(no. [%])*					0.832	<0.001	<0.001
	Normal or GI	616 (23.8)	97 (23.8)	39 (45.9)			
	GII	424 (16.4)	62 (15.2)	7 (8.2)			
	GIII, GIV, or OCD	1,553 (59.9)	249 (61.0)	39 (45.9)			

* BMI = body mass index; VR-12 MCS = Veterans RAND 12-Item Health Survey Mental Component Summary score; SD = standard deviation; KOOS = Knee injury and Osteoarthritis Outcome Score; GI, GII, GIII, and GIV = grades I through IV; and OCD = osteochondral defect.

† The values are given as the median with the 25th and 75th percentiles in parentheses.

‡ A complete tear is defined as tear extension from the center of the meniscus to the periphery, or extension through both the superior and inferior meniscal surfaces.

Regarding preoperative PROMs, clinically important and significant baseline differences between White and Black patients (p < 0.001 for each comparison) were again observed. White patients had higher median KOOS-pain scores (47.2 for White and 41.7 for Black patients), median KOOS-function scores (58.0 and 51.5, respectively), and median VR-12 MCS scores (55.9 and 48.4), indicating less severe knee symptoms and better mental health scores. Intraoperative findings showed differences by race with respect to the presence and location of complete meniscal tears but no difference in meniscal root involvement (p = 1.0) or highest encountered modified Outerbridge articular cartilage grade (p = 0.83).

Comparisons of patients of “other” race with White and Black patients are provided in Table I. Because of the small size of the “other” race group, these statistical comparisons should be interpreted with caution.

### Independent Associations with KOOS-Pain, KOOS-Function, and Articular Cartilage Grade

**Fig. 2:**
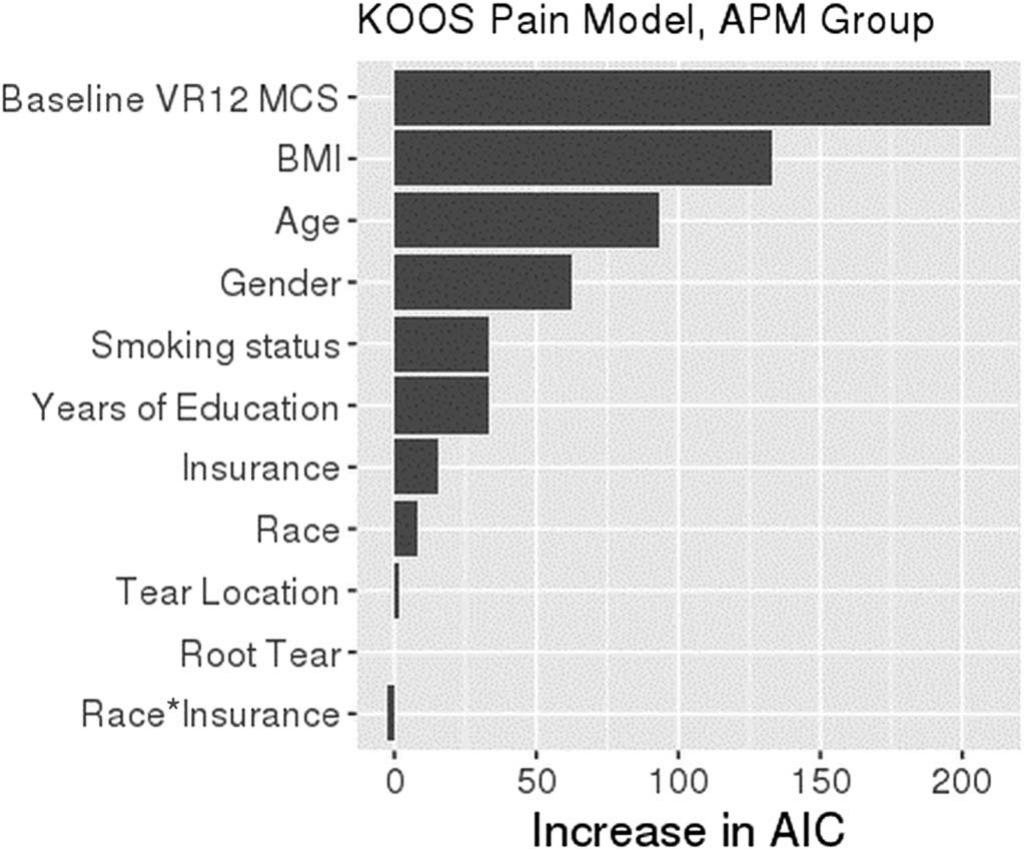
Variable importance plot for KOOS-pain. The relative importance of each variable in explaining KOOS-pain was ranked according to the increase in AIC (Akaike information criterion) on removal from the full model. When race or insurance is removed, the race-by-insurance interaction is removed as well. The asterisk indicates the interaction of the 2 variables. KOOS = Knee injury and Osteoarthritis Outcome Score, APM = arthroscopic partial meniscectomy, VR12 MCS = Veterans RAND 12-Item Health Survey Mental Component Summary score, and BMI = body mass index.

**Fig. 3:**
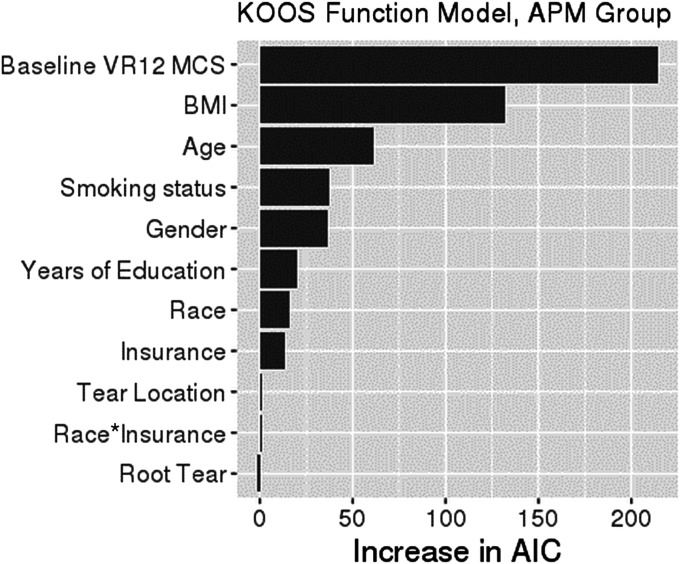
Variable importance plot for KOOS-function. The relative importance of each variable in explaining KOOS-function was ranked according to the increase in AIC (Akaike information criterion) on removal from the full model. When race or insurance is removed, the race-by-insurance interaction is removed as well. The asterisk indicates the interaction of the 2 variables. KOOS = Knee injury and Osteoarthritis Outcome Score, APM = arthroscopic partial meniscectomy, VR12 MCS = Veterans RAND 12-Item Health Survey Mental Component Summary score, and BMI = body mass index.

Black patients had an average KOOS-pain score that was 2.64 points (95% confidence interval [CI]: −4.73, −0.56; p = 0.013) lower than that for White patients after controlling for confounding variables (Table II). After accounting for confounding variables, a difference in KOOS-function was also observed between Black and White patients, although the magnitude of the difference was dependent on insurance status (Table III). Specifically, KOOS-function scores were a mean of 2.4 points (95% CI: −4.30, −0.45; p = 0.015) lower among commercially insured Black patients than among commercially insured White patients, and there was a nonsignificant difference (−7.56 points [95% CI: −16.3, 1.2]; p = 0.09) among Medicare-insured Black patients. However, patient race had a relatively small independent contribution to KOOS-pain or KOOS-function, with the 3 most important drivers of KOOS-pain and KOOS-function being baseline mental health (VR-12 MCS), BMI, and patient age (Figs. 2 and 3). All of the 3 most important drivers of KOOS-pain and KOOS-function in the multivariate analyses (Tables II and III) were unequally distributed between Black and White patients in the univariate analysis (Table I).

**TABLE II:** Linear Regression Model Results: KOOS-Pain*

Variable	Level	Estimate (95% CI)†	P Value‡
Age		−0.25 (−0.3, −0.2)	**<0.001**
Sex	Female (versus male)	−4.87 (−6.05, −3.68 )	**<0.001**
Race	Black (versus White)	−2.64 (−4.73, −0.56)	**0.013**
	Other (versus White)	−2.82 (−6.93, 1.3)	0.18
BMI		−0.53 (−0.61, −0.44)	**<0.001**
Years of education		0.6 (0.4, 0.8)	**<0.001**
Smoking status	Quit (versus never)	−2.98 (−4.33, −1.64)	**<0.001**
	Current (versus never)	−5.15 (−7.09, −3.21)	**<0.001**
Baseline VR-12 MCS		0.38 (0.33, 0.44)	**<0.001**
Insurance	Medicare (versus commercial)	2.15 (0.15, 4.16)	**0.035**
	Medicaid (versus commercial)	−4.84 (−7.84, −1.84)	**0.002**
Complete meniscal tear§	Yes (versus no)	2.2 (−0.65,5.05)	0.13
Complete tear location§	Medial (versus incomplete tear)	0.29 (−1.05, 1.63)	0.67
	Lateral (versus incomplete tear)	2.44 (0.55, 4.33)	**0.011**
	Both (versus incomplete tear)	−0.07 (−2.31, 2.17)	0.95
Race-insurance interaction#	Black-Medicare	−4.74 (−10.14, 0.65)	0.085
	Other-Medicare	7.64 (−4.37, 19.66)	0.213
	Black-Medicaid	−1.07 (−5.85, 3.72)	0.662
	Other-Medicaid	3.83 (−5.51, 13.17)	0.422

* KOOS = Knee injury and Osteoarthritis Outcome Score, BMI = body mass index, and VR-12 MCS = Veterans RAND 12-Item Health Survey Mental Component Summary score.

† This value represents the estimated change in KOOS-pain. As an example, the estimated change in KOOS-pain per 1 year increase in patient age is −0.25 points. The estimated difference in KOOS-pain among female versus male patients is −4.87 points.

‡ The values in bold indicate a significant difference.

§ A complete tear is defined as tear extension from the center of the meniscus to the periphery, or extension through both the superior and inferior meniscal surfaces.

# A significant interaction term between 2 variables indicates that the magnitude of the effect of 1 variable on the outcome depends on the second variable and vice versa.

**TABLE III:** Linear Regression Model Results: KOOS-Function*

Variable	Level	Estimate (95% CI)†	P Value‡
Age		−0.18 (−0.23, −0.14)	**<0.001**
Sex	Female (versus male)	−3.47 (−4.57, −2.38,)	**<0.001**
Race	Black (versus White)	−2.38 (−4.30, −0.45)	**0.015**
	Other (versus White)	−0.4 (−4.19, 3.4)	0.838
BMI		−0.48 (−0.56, −0.4)	**<0.001**
Years of education		0.43 (0.25, 0.61)	**<0.001**
Smoking status	Quit (versus never)	−3.04 (−4.29, −1.8)	**<0.001**
	Current (versus never)	−4.8 (−6.59, −3.01)	**<0.001**
Baseline VR-12 MCS		0.36 (0.31, 0.41)	**<0.001**
Insurance	Medicare (versus commercial)	1.88 (0.03, 3.73)	**0.046**
	Medicaid (versus commercial)	−3.94 (−6.71, −1.17)	**0.005**
Complete meniscal tear§	Yes (versus no)	0.75 (−1.89, 3.38)	0.578
Complete meniscal tear location§	Medial (versus incomplete tear)	0.84 (−0.4, 2.08)	0.185
	Lateral (versus incomplete tear)	1.78 (0.03, 3.53)	**0.046**
	Both (versus incomplete tear)	−0.92 (−2.98, 1.15)	0.385
Race-insurance interaction#	Black-Medicare	−7.06 (−12.04, −2.09)	**0.005**
	Other-Medicare	−1.91 (−13, 9.17)	0.735
	Black-Medicaid	−2.01 (−6.42, 2.4)	0.372
	Other-Medicaid	3.35 (−5.27, 11.97)	0.447

* KOOS = Knee injury and Osteoarthritis Outcome Score, CI = confidence interval, BMI = body mass index, VR-12 MCS = Veterans RAND 12-Item Health Survey Mental Component Summary score.

† This value represents the estimated change in KOOS-function. As an example, the estimated change in KOOS-pain per 1 year increase in patient age is −0.18 points. The estimated difference in KOOS-function among female versus male patients is −3.47 points.

‡ The values in bold indicate a significant difference.

§ A complete tear is defined as tear extension from the center of the meniscus to the periphery, or extension through both the superior and inferior meniscal surfaces.

# A significant interaction term between 2 variables indicates that the magnitude of the effect of 1 variable on the outcome depends on the second variable and vice versa.

**Fig. 4:**
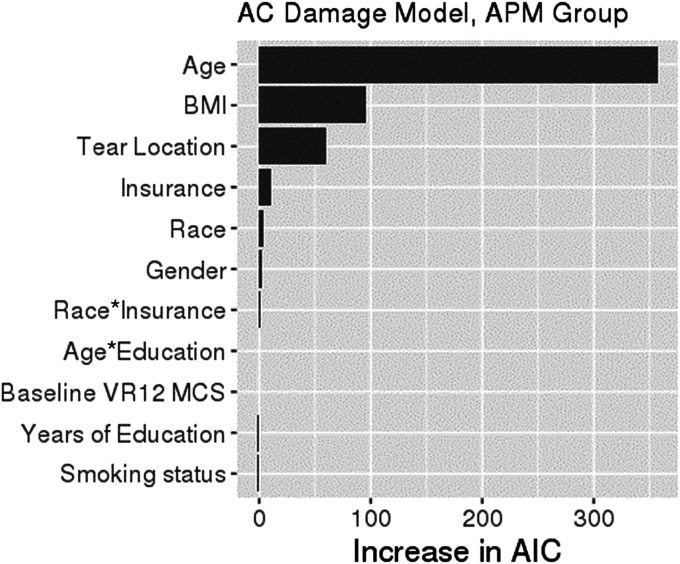
Variable importance plot for highest modified Outerbridge grade of articular cartilage (AC) damage. The relative importance of each variable in explaining the grade of articular cartilage damage was ranked according to the increase in AIC (Akaike information criterion) on removal from the full model. When race or insurance is removed, the race-by-insurance interaction is removed as well. The asterisk indicates the interaction of the 2 variables. KOOS = Knee injury and Osteoarthritis Outcome Score, BMI = body mass index, and VR12 MCS = Veterans RAND 12-Item Mental Component Summary score.

After controlling for confounding variables, the risk of higher-grade articular cartilage damage differed between White and Black patients, although the magnitude of the difference was dependent on insurance status. Compared with commercially insured White patients, commercially insured Black patients had 1.40-fold (95% CI: 1.06, 1.86; p = 0.019) greater odds of higher-grade articular damage, but there was no difference in risk among Medicare-insured Black patients (OR, 0.45 [95% CI: 0.13, 1.59]; p = 0.22). Race was a relatively unimportant predictor of articular cartilage damage, with the 3 most important drivers of higher-grade articular cartilage damage being patient age, BMI, and the location of a complete tear (Fig. 4). The 3 most important drivers of articular cartilage damage in the multivariate analysis (Table IV) were unequally distributed between Black and White patients in the univariate analysis (Table I).

**TABLE IV:** Proportional-Odds Regression Model Results: ICRS Articular Cartilage Grade*

Variable	Level	Odds Ratio (95% CI)†	P Value‡
Age		1.08 (1.07, 1.10)	**<0.001**
Sex	Female (versus male)	1.16 (1.00, 1.36)	0.056
Race	Black (versus White)	1.40 (1.06, 1.86)	**0.019**
	Other (versus White)	0.81 (0.52, 1.26)	0.346
BMI		1.06 (1.05, 1.08)	**<0.001**
Years of education		1.07 (1.03, 1.12)	**0.001**
Smoking status	Quit (versus never)	0.96 (0.80, 1.15)	0.645
	Current (versus never)	0.85 (0.66, 1.09)	0.194
Baseline VR-12 MCS		1.00 (1.00, 1.01)	0.194
Insurance	Medicare (versus commercial)	0.69 (0.53, 0.92)	**0.01**
	Medicaid (versus commercial)	1.23 (0.85, 1.77)	0.269
Complete meniscal tear location§	Medial (versus incomplete tear)	1.75 (1.47, 2.08)	**<0.001**
	Lateral (versus incomplete tear)	2.23 (1.75, 2.85)	**<0.001**
	Both (versus no tear)	2.53 (1.83, 3.49)	**<0.001**
Race-insurance interaction#	Black-Medicare	0.47 (0.23, 0.93)	**0.031**
	Other-Medicare	0.27 (0.26, 0.29)	**<0.001**
	Black-Medicaid	0.74 (0.40, 1.36)	0.333
	Other-Medicaid	0.46 (0.41, 0.53)	**<0.001**
Patient age-years of education interaction#		0.998 (0.997, 0.999)	**0.004**

* ICRS = International Cartilage Repair Society, CI = confidence interval, BMI = body mass index, and VR-12 MCS = Veterans RAND 12-Item Health Survey Mental Component Summary score.

† This value represents the increase in odds of a higher ICRS articular cartilage grade. As an example, the odds of a higher articular cartilage grade increase 1.08-fold per 1 year increase in patient age. The odds of a higher articular cartilage grade are 1.16-fold higher in female patients than male patients.

‡ The values in bold indicate a significant difference.

§ A complete tear is defined as tear extension from the center of the meniscus to the periphery, or extension through both the superior and inferior meniscal surfaces.

# A significant interaction term between 2 variables indicates that the magnitude of the effect of 1 variable on the outcome depends on the second variable and vice versa.

The results of the multivariate analyses are summarized in Table V. Because of the small size of the “other” race group, comparisons of “other” race patients with Black and White patients in the multivariate analyses should be interpreted with caution.

**TABLE V:** Summary of Significant Variables in Multivariate Analysis*

Variable	Level	KOOS-Pain†	KOOS-Function†	AC Damage†
Age		**X**	**X**	**X**
Sex	Female (versus male)	**X**	**X**	
Race	Black (versus White)	**X**	**X**	**X**
	Other (versus White)			
BMI		**X**	**X**	**X**
Years of education		**X**	**X**	**X**
Smoking status	Quit (versus never)	**X**	**X**	
	Current (versus never)	**X**	**X**	
Baseline VR-12 MCS		**X**	**X**	
Insurance	Medicare (versus commercial)	**X**	**X**	**X**
	Medicaid (versus commercial)	**X**	**X**	
Complete meniscal tear‡	Yes (versus no)			
Complete meniscal tear location‡	Medial (versus incomplete tear)			**X**
	Lateral (versus incomplete tear)	**X**	**X**	**X**
	Both (versus incomplete tear)			**X**
Race-insurance interaction§	Black-Medicare		**X**	**X**
	Other-Medicare			**X**
	Black-Medicaid			
	Other-Medicaid			**X**
Patient age-years of education interaction§				**X**

* KOOS = Knee injury and Osteoarthritis Outcome Score, BMI = body mass index, and VR-12 MCS = Veterans RAND 12-Item Health Survey Mental Component Summary score.

† The bold Xs indicate significant variables.

‡ A complete tear is defined as tear extension from the center of the meniscus to the periphery, or extension through both the superior and inferior meniscal surfaces.

§ A significant interaction term between 2 variables indicates that the magnitude of the effect of 1 variable on the outcome depends on the second variable and vice versa.

## Discussion

The principal findings of our study demonstrate that there is an imbalance between White and Black patients who undergo APM with regard to the factors most strongly associated with knee pain, knee function, and concomitant articular cartilage damage. After adjusting for multiple strong confounding factors, modest racial disparity persists, with generally worse knee pain, knee function, and articular cartilage damage among Black patients at the time of surgery. Although there are clear demographic differences between White and Black patients with orthopaedic conditions, prior racial disparity research in orthopaedics has rarely taken these factors into account^5-19^. This study represents a major advancement in our understanding of racial disparities in arthroscopically treatable knee conditions and highlights the fact that substantial confounding is present when comparisons are made between Black and White patients with regard to clinically relevant baseline factors (knee pain, knee function, and articular cartilage damage).

Among the most important confounders in the current study, the direction of confounding between Black and White patients was usually in favor of a better KOOS score or articular cartilage grade among White patients. Black patients had lower VR-12 MCS scores and higher BMI on average than white patients, both of which are associated with worse KOOS scores. Mental health has been shown to have a strong association with knee pain^28^, and correspondingly the VR-12 MCS was the strongest predictor of KOOS-pain and KOOS-function scores in this study. The suggested MCID in VR-12 MCS in the orthopaedic patient population is 5.99 points^29^, which is similar to the difference in VR-12 MCS scores between White (median, 55.9 points) and Black patients (median, 48.4 points) in the present study. The VR-12 MCS was calibrated for a score of 50 to represent the population norm (based on the U.S. population in 1990)^26^, suggesting that mental health scores among Black patients in the current study approximate normal values, but White patients in the current study had higher than normal values. The association between higher BMI and worse outcomes is logical, given that added mass can increase the forces experienced by the knee joint. This is supported in studies of middle-aged patients with osteoarthritis, in whom increased BMI resulted in worse symptoms and higher risk of osteoarthritis progression^30^. The difference in BMI between White (mean, 30.3 kg/m^2^) and Black patients (mean, 32.4 kg/m^2^) in this study is clinically important, as even 5 to 10 lb (2.3 to 4.5 kg) of weight loss can result in relief of knee symptoms^31^. Finally, patient age, which was a strong predictor of better KOOS scores as well as articular cartilage grade, was lower among Black patients (median, 50.0 years) than White patients (median, 54.0 years). Age had a strong influence on articular cartilage grade (Fig. 4), which explains in part why there was no observed difference in cartilage grade between Black and White patients in the univariate analysis, but a significant difference was observed after age adjustment in the multivariate analysis.

Years of education had a modest association with KOOS scores as well as articular cartilage health at the time of surgery, and there was an imbalance in educational level between White patients (6.7% with <12 years of education) and Black patients (15.9% with <12 years of education). The number of years of education has a positive association with health across a variety of conditions^32^. The possible reasons for the association between education and health are multifactorial; in addition to health insurance status, several relevant factors not specifically accounted for in the current study may be represented by years of education, including income, family background, knowledge, cognitive ability, and social networks^32^.

Current smoking rates were higher among Black patients (16.7%) than White patients (10.0%), and smoking status was associated with KOOS scores but not with articular cartilage health. There is limited literature on the effect of smoking in knee arthroscopic surgery, particularly with regard to PROMs^33^, although recent literature has suggested that smoking has an independent negative effect on knee ligament surgery and possibly knee articular cartilage surgery^7,33^. Smoking is associated with worse knee symptoms^34,35^, although it has been reported either to have no effect^36^ or to have a slight protective effect^37^ on the risk of developing knee osteoarthritis according to recent meta-analyses.

After adjustment for confounding factors, residual differences remained between Black and White patients with respect to KOOS scores and articular cartilage damage, all in the direction of worse knee symptoms and articular cartilage health among Black patients at the time of surgery. We cannot confirm the reasons why Black patients had worse symptoms and articular cartilage health, although we suspected that this may be because Black patients tend to wait longer to seek care for atraumatic knee pain. The trend observed in the knee arthroplasty literature, in which Black patients have more severe disease on presentation for knee arthroplasty, is that the Black versus White disparity in arthroplasty utilization is increasing with time^15^. Insurance status influenced outcomes in the current study, and there was a complex interaction among race, insurance status, and outcomes. We hypothesized that insurance status is an incomplete representation of economic status and that the resources available to Black versus White patients of equivalent insurance status are not equal.

There are several limitations to this study. This study was performed at a large hospital system in the midwestern United States. Patients in this geographic region may not be nationally representative. Because of the limited sample size of non-Black and non-White patients, we were only able to evaluate White-Black disparities with adequate statistical power. Surgeon recommendations for total knee arthroplasty do not appear to be influenced by patient race^38^; however, Black patients with knee osteoarthritis are less likely to consider total knee arthroplasty^39^ and have lower expectations than White patients with respect to surgical outcomes^40^. The influences of race on surgeon recommendations, patient consideration of surgery, or patient expectations after surgery have not been reported in the setting of knee meniscal tears but are potential sources of bias that could not be assessed in the current study. Finally, the effect of baseline racial disparities in knee symptoms and articular cartilage status on outcomes following partial meniscectomy cannot be determined with the current cross-sectional study.

### Conclusions

There are significant differences between Black and White patients presenting for APM with regard to several factors associated with worse knee pain and knee function and greater articular cartilage damage. After accounting for these confounding factors, a modest racial disparity remains with respect to knee pain, knee function, and cartilage damage. When controlling for the baseline risk factors between Black and White patients undergoing APM, there are small significant, but not clinically relevant, differences in baseline knee pain, function, and articular cartilage damage. The 3 most important risk factors for KOOS-pain and KOOS-function subscales are baseline mental health, BMI, and patient age. For articular cartilage damage, patient age, BMI, and complete tear location are the most important risk factors.
